# The neuropathological impact of COVID-19: a review

**DOI:** 10.1186/s42269-020-00478-7

**Published:** 2021-01-12

**Authors:** Ghadha Ibrahim Fouad

**Affiliations:** Department of Therapeutic Chemistry, National Research Centre, 33 El-Bohouth St., Dokki, Cairo, 12622 Egypt

**Keywords:** COVID-19, SARS-CoV-2, Neurological manifestations, Coronaviruses, Neuroinvasive potential

## Abstract

**Background:**

The Coronavirus disease 2019 (COVID-19) outbreak has become a challenging global issue after its emergence in December 2019. Due to the high adaptation of the virus, COVID-19 demonstrated a high transmission and infectivity potentials. Several studies demonstrated that severe acute respiratory syndrome coronavirus 2 (SARS-CoV-2) induce deleterious neurological manifestations through interacting with the central nervous system (CNS).

**Main body:**

The neuroinvasive potential of SARS-CoV-2 might contribute to its fatal behavior. Understanding the underlying mechanisms of this novel neuropathogen might contribute to the development of effective therapeutic strategies. The manifestations of neural damage in COVID-19 patients ranged from headache to severe encephalopathy and progression of preexisting neural disorders, it is speculated that neuroinvasion is strongly linked to the fatal respiratory dysfunction. The underlying neuropathological impact of emerging pneumonia (COVID-19) is still unclear.

**Conclusion:**

This review demonstrated the urgent need to understand the neuropathology of COVID-19, to manage the current borderless viral outbreak of SARS-CoV-2 and its comorbidities. Moreover, SARS-CoV-2 could be regarded as an opportunistic neuropathogen that affects several vital functions in the human body.

## Background

Coronavirus disease 2019 (COVID-19)-associated respiratory dysfunction in the upper respiratory tract was first reported to WHO as pneumonia of unknown etiology in Hubei Province, Wuhan City, China, on December 31, 2019 (WHO 2020; Thompson 2020). China was the first hub for the spreading of this emerging viral outbreak of SARS-CoV-2, causing a global pandemic. This challenging pandemic hit 188 countries and resulted in a huge toll of confirmed death cases. By the third week of October, the total number of confirmed cases reached 41,074,593 in the entire world, with 1,128,532 deaths; these data were provided by the Center for Systems Science and Engineering (CSSE) at Johns Hopkins University (JHU) (CSSE 2020). There is limited knowledge about mechanisms of COVID-19 infections; in addition, there is no effective therapeutic approach to manage this epidemic. Social distancing and lockdown in affected countries are the used strategies to limit the transmission of the viral infection.

**Fig. 1:**
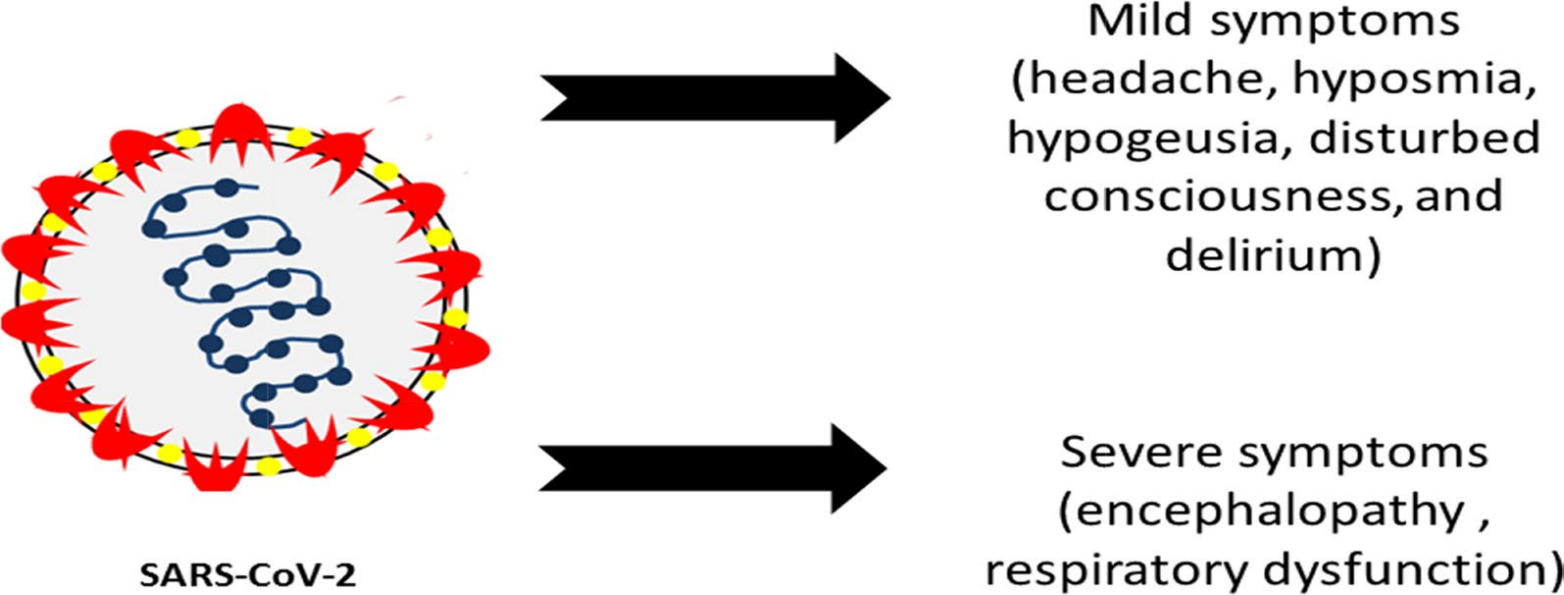
SARS-Cov-2 infection provokes mild symptoms and/or severe symptoms that could result in respiratory failure

The two most common and principal routes of transmission of SARS-CoV-2 are close contact with an asymptomatic carrier and respiratory virus-laden droplets. In addition, prolonged exposure to a high concentration of SARS-CoV-2 aerosols (exceeding the viral load) may contribute to the infection, especially in closed locations such as hospitals (Zhang et al. 2020). The principal route of SARS-CoV-2 transmission is the droplet contagion; the virus enters the intranasal and oral routes, infects the olfactory sensory neurons, and then invades the central nervous system (CNS) through the olfactory nerve; this explains the common sensory symptoms of hyposmia (a decreased sense of smell) and hypogeusia (a reduced ability to taste) (Li et al. 2020a). COVID-19 is a viral infection caused by severe acute respiratory syndrome coronavirus 2 (SARS-CoV-2). SARS-CoV-2 is a highly contagious and opportunistic virus that attacks the respiratory system. This infection ranged from asymptomatic infection (the incubation period) to respiratory failure, with clinical symptoms of headache, dry cough, and fever. The common clinical symptoms of COVID-19 include dry cough, fever, sore throat, dyspnea (shortness of breath), muscle soreness, fatigue, in addition to no notable improvement upon three days of treatment with antibiotics (Sun et al. 2020a). The case might get worse to acute respiratory distress syndrome (ARDS) that demands intensive care unit (Wang et al. 2020a) (Fig. 1). Moreover, SARS-CoV-2 demonstrated “extra-respiratory” actions in confirmed cases of COVID-19 (Lai et al. 2020), regarding the fact that coronaviruses are known to demonstrate symptoms of “multi-organ system damage” (Gulati et al. 2020). SARS-CoV-2 can attack several organs such as the heart, the liver, and the central nervous system (CNS). For example, some COVID-19 patients might suffer from a new-onset cardiac dysfunction (Paramasivam et al. 2020) or hepatic impairment (Wang et al. 2020b), or renal dysfunction (Li et al. 2020b). Politi et al. (2020) published the first report of human brain involvement in a COVID-19 patient. In addition, Moriguchi et al. (2020) reported a case of meningitis/encephalitis.

## Main text

### What are coronaviruses (CoVs)?

Coronaviruses (CoVs) are a large family of positive-sense, single-stranded (ss) RNA viruses, spherical or oval shaped, with an average diameter of 100 nm. CoVs possess surface spikes of viral membrane glycoproteins and demonstrate a typical crown-like shape by electron microscopy; the length of viral genome ranges between 26 and 32 kilobases (kb) (Schoeman and Fielding, 2019). Coronaviruses (CoVs) are classified into four genera, including α, β, γ, δ. "α- and β-" CoVs infect mammals, while "γ- and δ-" CoVs tend to infect birds. There are six identified CoVs as human-susceptible virus, among which β-CoVs, severe acute respiratory syndrome (SARS), and Middle East respiratory syndrome (MERS) lead to severe respiratory tract infections (Yin and Wunderink, 2018). SARS-CoV-2 is the seventh known CoV that can infect humans (Corman et al. 2019). SARS-CoV-2 was recognized as a β-coronavirus, that belongs to (subgenus *Sarbecovirus*, *Orthocoronavirinae* subfamily, *Coronaviridae* family), with 96.2% sequence homology with SARS-like coronavirus (bat-SL-CoVZC45), referring "bat" as the original host (Zhu et al. 2020; Guo et al. 2020; Lu et al. 2020). It was suspected that the bat is the natural and transmitting of SARS-CoV-2, regarding its similarity with SARS-CoV (Zheng 2020). The viral genome of CoVs encodes four main structural proteins to generate a structurally complete viral particle: the envelope (E) protein, membrane (M) protein, nucleocapsid (N) protein, and the spike (S) protein (Schoeman and Fielding, 2019). The "S protein" facilitates viral entry into the host cell and enables the fusion between the viral and host cell membranes (Kirchdoerfer et al. 2016; Zheng 2020).

It is speculated that SARS-CoV-2 has been introduced to humans by an unknown intermediate host before its genetic modification to transport from human to human. The infections of CoVs were mainly enzootic (i.e., limited only to their natural animal hosts) in birds and mammals. However, there is a sweeping deviation in the infectivity potential of CoVs by crossing the animal–human species barrier and infects humans. This was demonstrated in the first outbreak of SARS in China 2003, which was transmitted to 37 countries, and the second outbreak of MERS in Saudi Arabia in 2012 that was transmitted to 27 countries (Schoeman and Fielding 2019). COVID-19 represents the third presentation of a highly pathogenic and large-scale epidemic SARS-CoV-2 into the human population. Moreover, this high pathogenicity of SARS-CoV-2 might be ascribed to the asymptomatic infection of the virus that means that COVID-19 patients might transmit infection without the appearance of clinical symptoms, while tested positive for the presence of viral nucleic acids (Wang et al. 2020a); this could render the management of COVID-19 spreading more difficult.

### Neuropathological manifestations of COVID-19

Human coronaviruses (CoVs) are characterized by high mortality rate along with their ease of transmission (Schoeman and Fielding 2019). Human CoVs could invade both the respiratory system and the CNS; this family of viruses is featured by that neuroinvasion and neurotropism (Desforges et al. 2020). Several researchers reported the potential of SARS-CoV-2 to attack the CNS and result in neurodegeneration (Moriguchi et al. 2020; Asadi-Pooya and Simani 2020; Toscano et al. 2020; Xiang et al. 2020). In the initial phases of SARS-CoV-2 infection of the respiratory system, olfactory tract is the principal route for virus entry to the brain and the CNS (Zhang et al. 2020). CoV can invade the brain via the olfactory tract in the early stages of infection within seven days (Desforges et al. 2020); CoV infects nasal cells, accesses the brain and cerebrospinal fluid (CSF) through the olfactory nerve and olfactory bulb, and induces neural damage (Wu et al. 2020a).

Neurological symptoms involved headache, impaired consciousness, seizures, acute cerebrovascular disease, hyposmia (a decreased sense of smell), hypogeusia (a reduced ability to taste), and neuralgias (Montalvan et al. 2020). The occurrence of COVID-19-associated neurodegeneration might reach 40% (Mao et al. 2020); this neural damage might be caused by direct infection, hypoxia, and immune response (Wu et al. 2020a). Patients with a mild infection may suffer from headache, disturbed consciousness, and delirium (Wu et al. 2020a); neurological damage was more common in COVID-19 patients with severe infection (Lai et al. 2020) who suffer from multi-organ failure (MOF) and hypoxia (Asadi-Pooya and Simani 2020). Moreover, COVID-19 patients might develop situation of impaired mental function (Nicholls and Peiris 2005). Toxic and viral encephalopathy may occur because of severe hypoxia and viremia (Guo et al. 2020); the risk of incidence of encephalopathy increases in patients with "preexisting" neurological disorder (Gulati et al. 2020). This infectious (viral)/acute encephalopathy signifies acute infection associated with hypoxia, metabolic disorders, and systemic inflammation, resulting in brain dysfunction (Young 2013; Tauber et al. 2017). Encephalitis indicates the existence of pathogen-induced neuroinflammation in the brain parenchyma, with common systems of headache, high temperature, and consciousness disorder (Ellul and Solomon 2018). SARS-CoV-2 could be detected in CSF, using genome sequencing, and thus increased the possibility of viral encephalitis (Hung et al. 2003; Xiang et al. 2020). Postmortem examinations of the brain of COVID-9 patients have revealed partial neurodegeneration, tissue congestion, and edema (Wu et al. 2020a). There is a possible link between the neuroinvasive potential of this pathogen and the respiratory failure of COVID-19 (Li et al. 2020c). Additionally, CoVs can invade the CNS, where they may either induce neurodegeneration or remain latent (Matías-Guiu et al. 2020), regarding that if a virus gets in the CNS, it is difficult to get out (Reinhold and Rittner 2017). There could be a strong connection between the SARS-CoV-2 infection of the brain stem and the respiratory dysfunction, considering that the brain stem controls vital functions including respiration and maintenance of blood pressure; it was found that SARS-CoV-2 could infect brainstem through a synapse-connected route from the lungs (Zhang et al. 2020). From this perspective, clinicians should perform an early evaluation of COVID-19 patients for neurological symptoms to manage the COVID-19-associated neurological manifestations and prevent the development of viral infection into a neurodegenerative disorder (Wu et al. 2020a). Accordingly, there is an urgent need to perform "follow-up studies" in COVID-19-recovered patients, as well as to provide spiritual and medical support for this kind of patients, to assess both the mental status and the general health condition. Thus, psychiatric and psychological issues (e.g., cognitive rehabilitation) should be considered in both patients and medical staff (Balachandar et al. 2020), to mitigate the COVID-19-associated depression and stress (Li et al. 2020c; Politi et al. 2020). Therefore, more research is required to understand the neuroinvasion capacity of the virus and to prevent the neuropathological impact of COVID-19 in recovered patients (Balachandar et al. 2020).

#### The neuropathogenic mechanism of COVID-19:

**Fig. 2:**
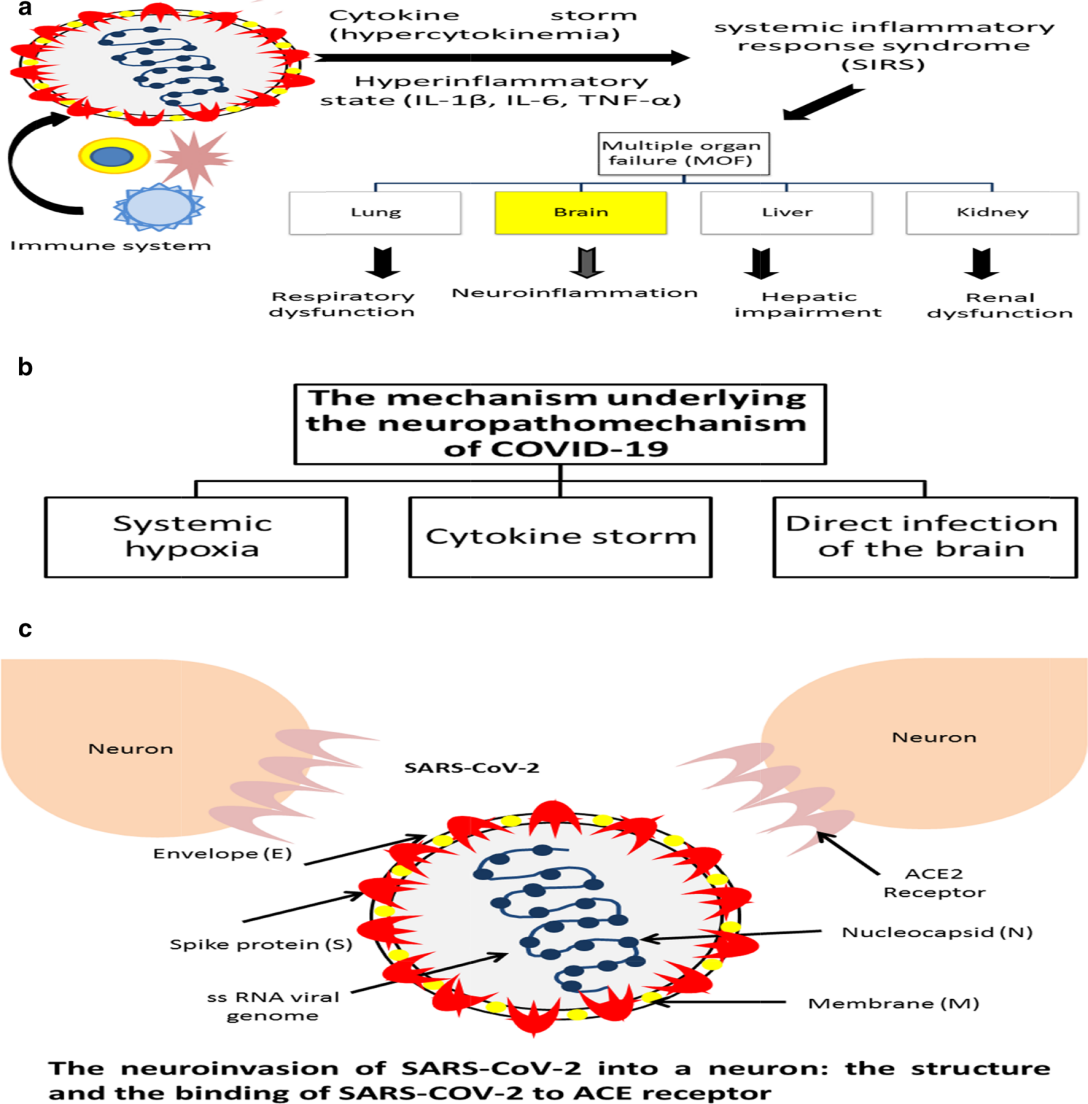
The mechanism of action of SARS-COV-2 as a neuropathogen; **a** The SARS-COV-2-induced cytokine storm followed by multiple organ failure (MOF), **b** the neuropathogenic mechanisms of SARS-COV-2 (systemic hypoxia, cytokine storm, direct invasion), **c** The structure of SARS-COV-2 and the mechanism of its invasion into a neural cells

The mechanism underlying the neuropathogenesis of COVID-19 might be attributed to three factors: the first factor is the occurrence of hypoxia (due to alveolar gas exchange disorders) to the brain. The SARS-COV-2-associated respiratory dysfunction could cause severe pneumonia that leads to systemic hypoxia, and the generation of toxic metabolites, due to increased anaerobic metabolism in the brain mitochondria, that finally results in brain interstitial edema, obstruction of cerebral blood flow (CBF), headache, and even a coma (Tu et al. 2020). Hypoxia might contribute to the development of the acute cerebrovascular disease such as acute ischemic stroke (Wu et al. 2020a). It was demonstrated that COVID-19 patients often suffer from severe hypoxia (Guo et al. 2020), which may cause subsequent neural damage (Fig. 2).

The second factor involves the interaction of immune response due to the high influx of inflammatory mediators and cytokines (e.g., interleukin 6) and the activation of macrophages and T-lymphocytes, thus promoting neuroinflammation (Mehta et al. 2020; Wu et al. 2020a). This might be attributed to the neurotropic nature and neuroinvasive potential of some viruses including human CoVs that result in activation of microglia, macrophages, or astrocytes in the nervous system (Soung and Klein 2018; Asadi-Pooya and Simani 2020). This neurotropic nature of this opportunistic pathogen might enhance a pro-inflammatory state (Li et al. 2004). Moreover, increased inflammatory cytokines and reduced lymphocytes are strongly correlated with the severity and progression of CoVs infection, including COVID-19 (Zhang et al. 2020). The SARS-COV-2-associated pneumonia is often linked to induced hyper-cytokinemia "cytokine storm" that result in generation of "hyper-inflammatory state" through uncontrolled over-production of multiple inflammatory cytokines and finally resulting in ARDS (Sun et al. 2020b). Therefore, this SARS-COV-2-induced systemic inflammatory response syndrome (SIRS) is a major player in the severity and mortality from this viral infection (Sun et al. 2020b). SIRS-mediated generation of free radicals and pro-inflammatory cytokines affects the microcirculation of the central and peripheral nervous system (Polidoro et al. 2020). Moreover, this "hyper-inflammatory state" might be strongly related to multiple organ failure (MOF) through resulting in auto-immunity as the high levels of inflammatory cytokines can attack other tissues (Science Mag 2020).

This phenomenon of "cytokine release syndrome (CRS)" can activate the endothelial cells and result in endothelial dysfunction and induce coagulopathy (Hay et al. 2017) and formation of blood clots that can break apart and deposit in the lungs, resulting in fatal "pulmonary embolism" or the brain and resulting in "stroke" in COVID-19 patients (Klok et al. 2020). This might be attributed to the abundance of angiotensin-converting enzyme 2 (ACE2) receptors in the endothelium and the blood lining (Klok et al. 2020).

CoVs infection (e.g., SARS and COVID-19) might be implicated in the development of a systemic inflammatory response syndrome (SIRS) that could be linked to multiple organ failure (MOF) (Wu et al. 2020b). COVID-19 patients might suffer from several endocrine and metabolic disruptions (*e.g.,* hypo-/hyper-natremia, hypo-/hyper-glycemia) and affect several organs causing kidney dysfunction, hepatic injury, and encephalopathy (Zubair et al. 2020). In severe COVID-19 patients, CRS participates to "toxic-metabolic encephalopathy" along with metabolic disruptions (Zubair et al. 2020).

Due to sequence homology with SARS-CoV, SARS-CoV-2 might simulate the behavior of SARS and MERS and induce neurodegeneration in the respiratory center in the medulla via enhancing the release of multiple inflammatory cytokines IL-1, IL-6, and TNF-α (Montalvan et al. 2020), regarding that IL-6 is strongly associated with the severity of viral infection (Wan et al. 2020). This CRS of SARS-CoV-2 infection is currently linked to the occurrence of acute cerebrovascular disorder (Huang et al. 2020; Chen et al. 2020; Mehta et al. 2020). One recent case report described Guillain-Barré syndrome (GBS), an immune-mediated neurological disorder, in COVID-19 patient, the neurological symptoms began two weeks after infection with SARS-COV-2 (Sedaghat and Karimi 2020); this demonstrated the potential of SARS-COV-2 to enhance auto-immunity, as the virus induces CRS and the uncontrolled cytokines attack the intact organs. Moreover, it has been assumed that SARS-CoV-2 might contribute to the alteration of the immune response of patients with multiple sclerosis (MS); this interaction might present a potential relation between SARS-CoV-2 and MS (Duffy and O'Reilly 2016). Moreover, viral infection-associated immune response could result in elevated serum levels of pro-inflammatory cytokines that result in skeletal muscle damage in COVID-19 patients (Ahmad and Rathore 2020).

The third factor is the direct infection of the brain; neuroinfections starts when SARS-CoV-2 passed through the nasal cavity, followed by the olfactory nerve, and finally the virus gained entry to the brain (Koyuncu et al. 2013). Using electron microscope, neurons were found to have viral particles entrapped in dilated vesicles (Zubair et al. 2020). Actually, the viral genome and proteins of some viruses could be detected in CSF or brain, indicating that viruses can directly invade the nervous system and cause neural damage (Koyuncu et al. 2013; Leber et al. 2016). It was evidenced that CoVs can penetrate the brain by spillage from the lungs into the blood "systemic vascular dissemination" or by trans-neuronal passage from nerve endings in the nasal epithelium or through spreading from airway receptors to "medullary cardio-respiratory centers" (Desforges et al. 2020; Natoli et al. 2020). Another suggested mechanism of infection, known as "Trojan horse mechanism," demonstrated that viral particles could infect immune cells (*e.g.,* leukocytes), which causes systemic inflammation and disrupts the permeability of the blood–brain barrier (BBB) and allows the entry of more infected cells or even the virus particles into the brain (Zubair et al. 2020). This factor was supported by the fact that about 90% of COVID-19 patients could not breathe and might demonstrate neurological manifestations, such as headache, seizure, and impaired consciousness; this neuroinvasive capacity of SARS-CoV-2 might contribute to the fatal respiratory failure (Li et al. 2020c). Furthermore, this explains why some infected patients demonstrate clear neurological symptoms such as cerebrovascular complications that could get worse into respiratory failure, and why other do not (Li et al. 2020a). Older COVID-19 patients, who developed cerebrovascular disorder, possess high levels of D-dimer and C-reactive protein (CRP) and are at a hyper-coagulation condition (Zubair et al. 2020).

In addition, the circulating viral RNA in the plasma might directly infect the endothelial cells of BBB (Li et al. 2020c). It was demonstrated that SARS-CoV-2 infection might affect the endothelial cells and result in "systemic vascular endotheliitis" that enhance disruption of vascular homoeostasis, vasoconstriction, organ ischemia, and edema, and might create a "pro-coagulate state"; this endotheliitis might be involved in the occurrence of cerebrovascular stroke and coagulopathy (Román et al. 2020; Varga et al. 2020). Viral infection of SARS-CoV-2 might increase the incidence of "thromboembolism" in association with hypoxia and intravascular coagulation (Román et al. 2020). Strokes, either ischemic or hemorrhagic, are regarded as complications of SARS-CoV-2; binding of SARS-CoV-2 to ACE2 enhances arterial hypertension that could cause "intracerebral hemorrhage" (Guan et al. 2020) (Fig. 1).

#### Interaction of SARS-CoV-2 and angiotensin-converting enzyme 2 (ACE2)

In this review, we demonstrate the possible neuropathogenic route by which SARS-CoV-2 may penetrate based on previous researches of other respiratory viruses such as SARS and MERS (Li et al. 2012, 2016); COVID-19-associated pneumonia shares similar pathogenesis with that of SARS or MERS (Song et al. 2019). SARS-CoV-2 shares "sequence homology" with SARS-CoV and a bat coronavirus (Gorbalenya et al. 2020); the "genetic similarity" between SARS-CoV-2 and its cousin SARS-CoV reached 79.5%, while its similarity to bat-coronavirus is 96% (Wu et al. 2020b). However, SARS-CoV-2 is characterized by the nucleotide alterations in the spike (S) protein and its receptor-binding domain (RBD) (Kannan et al. 2020). Moreover, SARS-CoV-2 uses the same receptor as SARS-CoV for viral invasion into human host cells (Li et al. 2020c). Due to the common features and high sequence homology between SARS-CoV and SARS-CoV-2, SARS-CoV-2 might exhibit the same invasion potential (Li et al. 2020c), through using the same cellular receptor (ACE2), which facilitates the entry of SARS-CoV-2 into human host cells. The high expression and notable abundance of ACE2 in almost organs including lungs, lung parenchyma, vascular endothelia, brain, and endothelial cells facilitate the spreading of circulating SARS-CoV-2 via the circulatory system (Hamming et al. 2004). This supports the deleterious effects of SARS-CoV-2 on whole body systems and its involvement in MOF, despite the fact that the lung is "the principal battle zone" (Zhang et al. 2020). The increased expression of ACE2 in the respiratory tract facilitates entry of SARS-CoV-2 (Rothan and Byrareddy 2020). Once in the systemic circulation, the neurotrophic SARS-CoV-2 invades the neural tissue, where it binds with ACE2 receptors in the endothelium of capillaries (Baig et al. 2020). More interestingly, ACE2 is highly expressed in the two main regions responsible for the regulation of the respiratory cycle, ventrolateral medulla, and the nucleus of the *tractus solitarius* (Montalvan et al. 2020); the expression of ACE2 marks tissues susceptible to viral infection. A study by Netland et al. (2008) showed that the olfactory nerve was the main invasion route for the virus to invade the brain of SARS-CoV-infected transgenic mouse model of human ACE2. SARS-CoV-2 invades the host cells via direct interaction of its spike, a surface glycoprotein, with ACE2 (Gheblawi et al. 2020; Li 2016). Actually, after interacting with ACE2 in the endothelium of cerebral blood vessels, SARS-CoV-2 could result in disruption of BBB, thereby disrupting the permeability of BBB, facilitating the viral neuroinvasion into CNS; in addition, the ACE2 abundance in the brain enhances the neuroinfections (Li et al. 2020d; Mao and Jin, 2020). Moreover, the high pathogenicity and powerful spreading potential of SARS-CoV-2 might be attributed to the high binding affinity of the spike protein surface unit 1 to ACE2 (Wrapp et al. 2020; Zou et al. 2020) (Fig. 2).

### Future research and directions

Currently, there are no FDA-approved drugs for COVID-19; it is vital to find antiviral drugs or virucidal agents or vaccines to treat this novel viral infection. Several antiviral drugs are regarded as possible candidates (Guo et al. 2020). COVID-19 patients should receive antiviral drugs or virucidal agents to modulate the immune response (Li et al. 2020c; Zhang et al. 2020). Therefore, to design a therapeutic strategy for COVID-19 patient, it is mandatory to provide symptomatic treatment, to assess the vital functions of different organs, and to receive the required care. These precautions aimed at breaking this vicious circle of infection to reduce the risk of inflammatory events and promote the outcomes. Besides, the neuroinvasive potential and the neurotropic nature of SARS-CoV-2 should be investigated to apply an effective therapeutic approach that targets the molecular disruption of the neural system. Socially, it is mandatory to prepare the public awareness for the emergence of future pandemics and to develop virus prevention measures including social distancing, smart working, and promoting hygienic manners and to delay the onset of outbreaks and the incidence of the peak.

## Conclusion

The neurological manifestations appeared in the severe cases of viral infection of SARS-CoV-2; however, we could assume that neurological manifestations might start from the incubation period of the viral infection in the form of headache, dizziness, and alterations in mental status. To sum it all up, there could be notable neurochemical interplay between SARS-CoV-2 and CNS. Therefore, careful clinical assessment of the patient should involve the neurological manifestations, particularly those hospitalized patients, to alleviate the potential mortality of neuroinfection-associated respiratory failure. There is still relatively limited availability of data about several aspects of COVID-19 to enable tackling the disease. Defining the COVID-19-associated neurological disorders is of the greatest importance to design an effective therapeutic approach, which targets the neurotropic nature of SARS-CoV-2, for neuroprotection.

## Data Availability

Not applicable.

## Abbreviations

COVID-19: Coronavirus disease 2019
SARS: Severe acute respiratory syndrome
MERS: Middle East respiratory syndrome
WHO: World Health Organization
CNS: Central nervous system
ARDS: Acute respiratory distress syndrome
CoVs: Coronaviruses
CSF: Cerebrospinal fluid
SIRS: Systemic inflammatory response syndrome
MOF: Multiple organ failure
CRS: Cytokine release syndrome
ACE2: Angiotensin-converting enzyme 2
TNF-α: Tumor necrosis factor alpha
IL-6: Interleukin 6
GBS: Guillain–Barré syndrome
MS: Multiple sclerosis
BBB: Blood–brain barrier
CRP: C-reactive protein
